# Management of Anorectal Malformations at a Tertiary Hospital in Tanzania: A Retrospective Cross-Sectional Study

**DOI:** 10.7759/cureus.113020

**Published:** 2026-07-20

**Authors:** Mohamed Ibrahim, Ahmed Salman, Elkhider Babiker

**Affiliations:** 1 Urology, Queen Elizabeth Hospital Birmingham, Birmingham, GBR; 2 Diabetes and Endocrinology, Heartlands Hospital, Birmingham, GBR; 3 Paediatrics, Rwanda Military Hospital, Kigali, RWA

**Keywords:** anorectal malformations, colostomy, management patterns, paediatric surgery, staged anoplasty

## Abstract

Background

Anorectal malformations (ARMs) are among the most common congenital gastrointestinal anomalies and encompass a spectrum of defects requiring individualized, often staged, surgical management. Delayed diagnosis, limited access to specialist paediatric surgical services, and loss to follow-up remain significant challenges in low- and middle-income countries. Despite being a major tertiary referral centre, published data describing the presentation and management of ARMs in Tanzania are limited. This study aimed to describe the demographic characteristics, patterns of presentation, anatomical subtypes, surgical management, and treatment completion of children with ARMs managed at Muhimbili National Hospital (MNH), Tanzania.

Methods

A retrospective review of 100 ARM cases managed at MNH in 2023 was conducted. This study featured a 12-month study period. Data included demographics, ARM type, timing of diagnosis, surgical procedures, and treatment completion.

Results

The mean age at presentation was 52.4 weeks; 56% were female. The mean management time was 113 weeks. Most patients (84%) were diagnosed within the first two weeks of life. Imperforate anus without fistula (38%) and vestibular fistula (37%) were the most common types. Colostomy was performed in 88% of patients, mainly divided colostomy (86%). Posterior sagittal anorectoplasty (PSARP) was the predominant definitive repair (60%). Only 49% completed all stages of management, with ARM type significantly associated with completion (p = 0.049).

Conclusion

Management practices at MNH follow standard approaches, but prolonged treatment duration and low completion rates highlight the complexity of the malformations, along with the need for stronger follow-up systems and adequate, early diagnosis.

## Introduction

Anorectal malformations (ARMs) represent a spectrum of congenital anomalies affecting the distal gastrointestinal tract and are frequently associated with genitourinary and musculoskeletal abnormalities [1]. The global incidence is estimated at approximately one in 4,000-5,000 live births [1,2]. Advances in classification systems, surgical techniques, and perioperative care have improved survival and functional outcomes, particularly in high-resource settings [2,3].

The Krickenbeck classification provides a standardised framework for describing ARMs according to anatomical characteristics and fistula type, facilitating comparison across studies and guiding management decisions [4,5]. Posterior sagittal anorectoplasty (PSARP), first described by DeVries and Peña, remains the principal technique for definitive repair in most patients [6].

Management commonly involves a staged approach, particularly for intermediate and high malformations, with formation of a colostomy followed by definitive repair and subsequent colostomy closure [3,7]. Despite advances in surgical care, long-term functional outcomes remain variable, and access to timely, comprehensive management differs substantially across regions [8].

In low- and middle-income countries, delayed presentation, limited access to paediatric surgical services, and loss to follow-up continue to pose major challenges to optimal care [9]. Studies from sub-Saharan Africa have reported late presentation and incomplete treatment pathways among children with ARMs [10,11]. However, data from tertiary centres in East Africa remain limited. This study aims to describe the presentation and management of children with ARMs at a tertiary referral hospital, including progression through treatment and completion of planned surgical care, and to contextualise these findings within existing regional and international literature.

## Materials and methods

Study design and setting

This was a retrospective observational study conducted at Muhimbili National Hospital (MNH), a tertiary referral hospital in Dar es Salaam, Tanzania. MNH serves as the national referral centre for paediatric surgical conditions and receives patients from across the country. 

Study population

The study included all children diagnosed with ARMs who were managed at MNH during the year 2023. Patients were identified through hospital records, including admission registers, operative logs, and patient files. 

Inclusion and exclusion criteria

All patients with a confirmed diagnosis of ARM who presented to MNH and were managed during the study period were included in the analysis. Patients with incomplete records that precluded extraction of key study variables were excluded.

Data collection

Data were collected retrospectively using a structured data extraction form. Variables collected included demographic characteristics, age at diagnosis, sex, type of ARM, associated anomalies, initial management strategy, definitive surgical procedure, use of colostomy, postoperative care including anal dilatation, and treatment completion status at the end of the study period. This study was reported in accordance with the STROBE (Strengthening the Reporting of Observational Studies in Epidemiology) guidelines.

Classification of ARMs

ARMs were classified according to the Krickenbeck classification system based on anatomical findings and the presence or absence of fistulae.

Outcome measures

The primary outcomes were patterns of presentation and management of ARMs. Secondary outcomes included treatment completion status at the end of the study period, defined as completion of all planned stages of management, ongoing management, or failure to complete treatment.

Ethical considerations

Ethical approval for this study was obtained from the Muhibili National Hospital research department (Ref no: MNH/CRTCU/Perm/2024/072). As this was a retrospective review of routinely collected clinical data, the requirement for informed consent was waived. Patient confidentiality was maintained throughout the study, and no identifiable information was included. Additionally, Ethical approval was received from the dean of the University of Medical Sciences and Technology.

## Results

Study population

**Figure 1:**
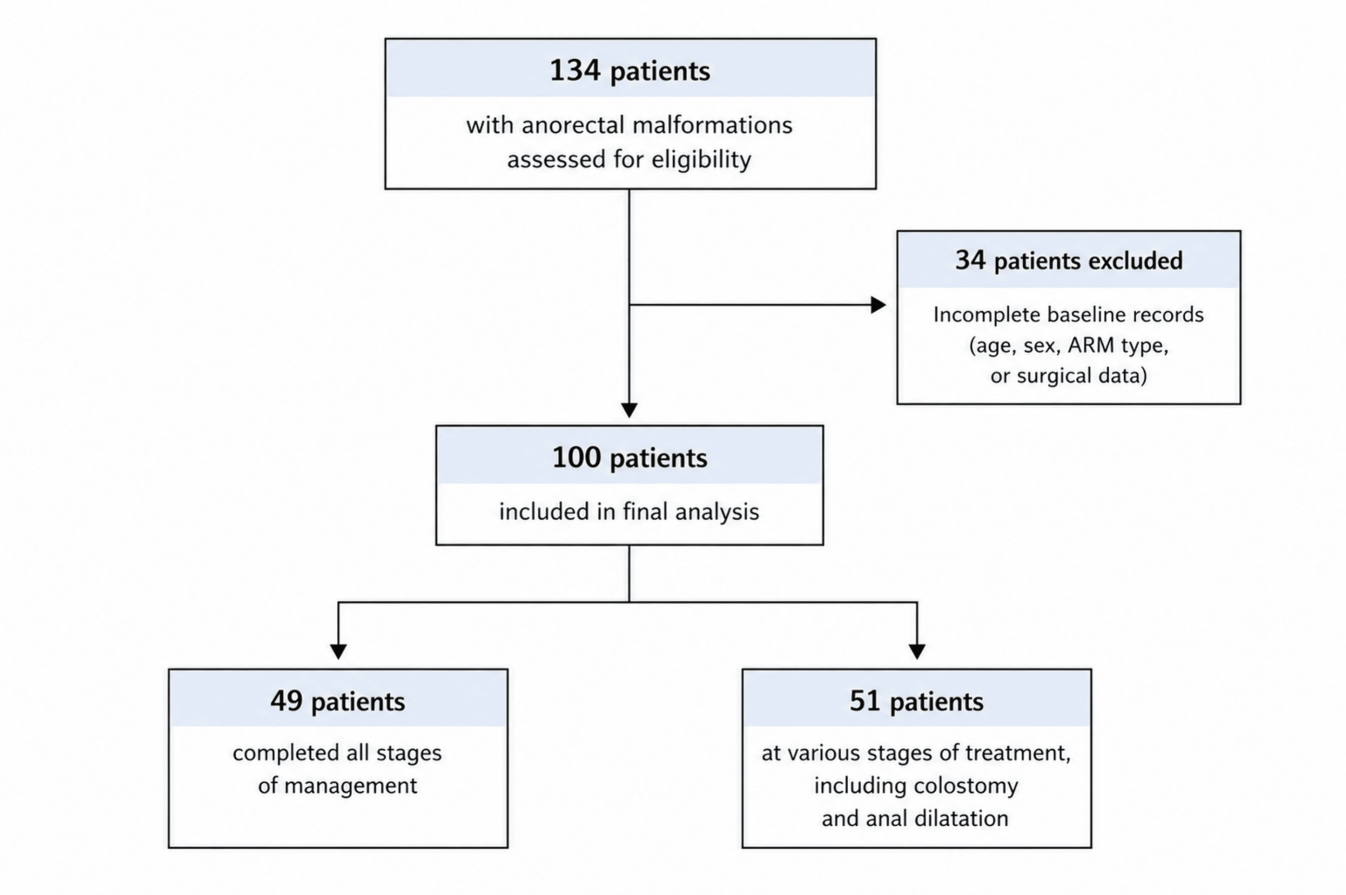
Patient flow diagram Patient flow diagram showing study eligibility, exclusions, final inclusion, and management status of patients with anorectal malformations.

A total of 134 patients with anorectal malformations were assessed for eligibility during the study period. After exclusion of records lacking essential baseline data, 100 patients were included in the final analysis. Patient inclusion and treatment status are illustrated in Figure 1.

Demographic characteristics

The demographic characteristics of the cohort are summarised in Table 1. The mean age at presentation was 52.4 weeks (range: 0-364 weeks). Females accounted for 56% of the cohort, while males constituted 44%. The majority of patients (84%) were diagnosed within the first two weeks of life, with a mean age at diagnosis of 14.4 weeks.

**Table 1:** Summary of patient demographics, clinical characteristics, surgical management, and treatment outcomes

Characteristic	Variable	Value
Patient demographics	Mean age at presentation	52.4 weeks (range: 0–364)
	Female sex	56%
	Male sex	44%
	Diagnosed within ≤2 weeks of life	84%
	Mean age at diagnosis	14.4 weeks
Timing of diagnosis	At birth	44%
	Within first 2 weeks	40%
	After 2 weeks from birth	16%
Type of anorectal malformation	Imperforate anus without fistula	38%
	Vestibular fistula	37%
	Cloaca	11%
	Rectourethral fistula	5%
	Rectal atresia	7%
	Rectal stenosis	2%
Type of colostomy	Divided (double-barrel)	86%
	Loop	11%
	End	1%
	Not specified	2%
Type of anorectoplasty	Posterior sagittal anorectoplasty (PSARP)	60%
	Anterior sagittal anorectoplasty (ASARP)	14%
	Abdominal pull-through	5%
	Primary repair	10%
	Not specified	11%
Management status at the last follow-up	Completed all stages	49%
	Still at colostomy stage	20%
	Undergoing dilatation	31%
Duration of management	Mean total duration (completed cases)	113 weeks
	Range	1–301 weeks
	Mean time to colostomy closure	131 weeks

Timing of diagnosis

The timing of diagnosis is presented in Table 1. Diagnosis was made at birth in 44% of patients. An additional 40% were diagnosed within the first two weeks of life, while 16% were diagnosed after two weeks of age.

Types of anorectal malformations

The distribution of anorectal malformations according to classification is shown in Table 2. The most common anorectal malformation was imperforate anus without fistula (38%), followed closely by vestibular fistula (37%). Other malformations included cloaca (11%), rectal atresia (7%), rectourethral fistula (5%), and rectal stenosis (2%). A statistically significant association was observed between ARM type and sex (p < 0.001).

**Table 2:** Association between sex and type of anorectal malformation

Type of anorectal malformation	Male, n (%)	Female, n (%)	Total, n (%)	Test statistic	p-value
Vestibular fistula	0 (0.0)	37 (100.0)	37 (37.0)		
Rectourethral fistula	5 (100.0)	0 (0.0)	5 (5.0)		
Imperforate anus without fistula	33 (86.8)	5 (13.2)	38 (38.0)		
Rectal atresia	4 (57.1)	3 (42.9)	7 (7.0)		
Rectal stenosis	2 (100.0)	0 (0.0)	2 (2.0)		
Cloaca	0 (0.0)	11 (100.0)	11 (11.0)		
Total	44 (44.0)	56 (56.0)	100 (100.0)	χ² = 75.420	<0.001*

Surgical management

Colostomy characteristics are summarised in Table 1. Colostomy formation was the most frequent initial surgical intervention. The majority of colostomies were divided (double-barrel) colostomies (86%), while loop colostomies accounted for 11%, and end colostomies for 1%. In 2% of cases, the colostomy type was not specified.

Definitive surgical procedures are presented in Table 1. Definitive surgical repair was most commonly performed using PSARP in 60% of patients. Anterior sagittal anorectoplasty (ASARP) was performed in 14%, abdominal pull-through in 5%, and primary repair in 10%. In 11% of cases, the type of definitive procedure was not documented.

Treatment completion

The treatment completion status is summarised in Table 3. At the time of the last follow-up, 49% of patients had completed all planned stages of management, 20% remained at the colostomy stage, while 31% were undergoing postoperative anal dilatation. A statistically significant association was observed between ARM type and treatment completion status (p = 0.049), as shown in Table 3.

**Table 3:** Association between the type of anorectal malformation and management completion status

Type of anorectal malformation	Management completed, n (%)	Management incomplete, n (%)	Total, n (%)	Test statistic	p-value
Vestibular fistula	25 (67.6)	12 (32.4)	37 (37.0)		
Rectourethral fistula	2 (40.0)	3 (60.0)	5 (5.0)		
Imperforate anus without fistula	17 (44.7)	21 (55.3)	38 (38.0)		
Rectal atresia	2 (28.6)	5 (71.4)	7 (7.0)		
Rectal stenosis	1 (50.0)	1 (50.0)	2 (2.0)		
Cloaca	2 (18.2)	9 (81.8)	11 (11.0)		
Total	49 (49.0)	51 (51.0)	100 (100.0)	χ² = 9.554	0.049*

Duration of management

The duration of management is presented in Table 4. Among patients who completed treatment, the mean total duration of management was 113 weeks, with a range of one to 301 weeks. The mean time to colostomy closure was 131 weeks. No statistically significant association was found between the type of definitive surgical procedure and overall duration of management (p > 0.05).

**Table 4:** Association between the type of anorectoplasty and time taken for management in weeks

Type of anorectoplasty	n (%)	Mean rank	Statistical test	Test statistic	p-value
Posterior sagittal anorectoplasty (PSARP)	33 (67.3)	22.65			
Anterior sagittal anorectoplasty (ASARP)	11 (22.4)	30.5			
Abdominal pull-through	2 (4.1)	33.25			
Not specified	3 (6.1)	25.17			
Total	49 (100.0)	—	Kruskal–Wallis test	H = 3.189	0.363

## Discussion

This study describes the presentation and management of children with ARMs at a tertiary referral centre. The spectrum of ARMs observed in this cohort is broadly comparable to patterns reported in international series [1,2,4].

A significant finding in this study is the delay in diagnosis and presentation, with many patients presenting beyond the neonatal period. Early diagnosis of ARMs is critical to prevent complications, such as bowel obstruction, sepsis, and electrolyte disturbances [12]. Delayed presentation has similarly been reported in studies from Tanzania and Nigeria, where limitations in neonatal examination, referral systems, and access to specialised care contribute to late diagnosis [10,11]. The findings of this study are consistent with these reports and reflect ongoing systemic barriers within resource-limited settings.

The majority of patients in this cohort required a staged surgical approach, commonly beginning with colostomy formation prior to definitive repair. This approach aligns with established management principles for complex and high ARMs [3,7]. However, a substantial proportion of patients did not complete all stages of management. Loss to follow-up and incomplete treatment pathways remain major challenges in low- and middle-income countries and have been widely reported in sub-Saharan Africa [9-11]. Factors such as financial constraints, travel distance, and limited caregiver understanding of the staged nature of treatment may contribute to this attrition.

Colostomy-related care and prolonged intervals between surgical stages may also influence treatment completion. Although colostomy remains an essential component of management for selected patients, prolonged diversion has been associated with stoma-related complications and increased caregiver burden [7,8]. In high-resource settings, structured follow-up and multidisciplinary support facilitate timely progression through surgical stages and long-term functional assessment [8]. In contrast, the challenges identified in this study underscore the difficulties of maintaining continuity of care in resource-limited environments.

Overall, the findings of this study are consistent with reports from other African centres and highlight persistent disparities in the management of ARMs compared with high-income settings. Addressing delays in diagnosis, strengthening referral pathways, and improving follow-up mechanisms may enhance treatment completion and outcomes in similar contexts.

This study has several limitations. Its retrospective design relied on the accuracy and completeness of medical records, and some patients had incomplete follow-up data. Functional outcomes such as continence and quality of life were not assessed, as these require long-term follow-up beyond the scope of the present analysis. Nevertheless, the study has several strengths, including a relatively large sample size, use of a standardised ARM classification system, and inclusion of patients at all stages of management, providing a realistic representation of clinical practice.

## Conclusions

Management of ARMs at Muhimbili National Hospital largely adheres to internationally accepted surgical principles, with most patients diagnosed early and treated using established operative techniques, particularly staged repair with colostomy and posterior sagittal anorectoplasty where appropriate. However, the prolonged duration of management and the low rate of treatment completion observed in this cohort highlight ongoing challenges in delivering comprehensive care for children with ARM in a resource-limited setting. These findings suggest that, despite appropriate surgical management, barriers to continuity of care remain significant. Strengthening referral pathways, improving caregiver education, enhancing follow-up systems, and optimising coordination of staged surgical management may improve treatment completion and overall patient outcomes. Further prospective, multicentre studies with long-term functional follow-up are needed to better evaluate outcomes and identify strategies to improve the management of ARMs in Tanzania and similar low-resource settings.
